# Correlations between eyelid tumors and tear lipocalin, lysozyme and lactoferrin concentrations in postmenopausal women

**Published:** 2015

**Authors:** I Careba, D Gradinaru, A Chiva, M Totir, R Ciuluvica, S Gradinaru, E Ungureanu

**Affiliations:** * Ophthalmology Department, “Carol Davila” University of Medicine and Pharmacy, Bucharest, Romania; ** Anatomy Department, “Carol Davila” University of Medicine and Pharmacy, Bucharest, Romania; *** Obstetrics and Gynecology Department, Regina Maria - Private health care network; **** Ophthalmology Department, Regina Maria - Private health care network; ***** University Emergency Hospital Bucharest, Romania

**Keywords:** Dry eye, tear lipocalin, lysozyme, lactoferrin, lipocalin

## Abstract

**Rationale:** Common ophthalmological problems are found in patients with eyelid tumors and include ocular surface diseases, such as dry eyes, eyelid disorders, excessive tearing and ocular inflammation.

**Objective:** The potential correlation between the symptomatology, tear break-up time (TBUT) and lipocalin, lactoferrin and lysozyme concentrations in the tear film were investigated in a group of symptomatic dry-eyed postmenopausal (PM) women compared to age-matched controls, considering the patients with eyelid tumors.

**Methods and Results:** 66 females were divided into two groups of 33 females each, one group having dry eye (DE) and one asymptomatic group (non-dry eye) (NDE), based on their responses to the OSDI questionnaire, TBUT and Schirmer test evaluation. Tears were collected via capillary tubes. Tear lysozyme, lactoferrin and lipocalin concentrations were determined via electrophoresis and the results for patients with or without eyelid tumors were compared. The results revealed significant differences in lysozyme concentration between patients with or without eyelid tumors in the DE group (p = 0.004). Lower levels for TBUT and lactoferrin in the DE group were also found, compared to the NDE group for eyelid tumors patients. Tear lipocalins were in the same range in both groups.

**Discussion:** Within a PM population, some components of the tear film were found to be at lower levels in patients with eyelid tumors, compared to patients without this pathology, which resulted in the development of DE or in the enhancement of the symptoms of an existing DE.

**Abbreviations:** DE = dry eye disease, NDE = non-dry eye, EGF = epidermal growth factor, ELISA = Enzyme-linked immunosorbent assay, SDS = sodium dodecyl sulfate, MMP = Matrix metalloproteinases.

## Introduction

One of the most common conditions seen by the ophthalmologists worldwide is dry eye syndrome (DE), with a prevalence of 7.4% and 33.7% [**1**]. DE affects women more than men, particularly elderly patients [**2**–**5**]. The risk factors include ageing, menopause, androgen deficiency and oral contraceptive treatment. They interfere with tear secretion, Meibomian gland function and goblet cell density leading to dry eye syndrome [**1**]. New tear film biomarkers are necessary to be established, in order to early and accurately diagnose DE.

The biochemical changes discovered in DE usually appear before the clinical signs and symptoms. This is the reason why the early diagnosis is very important. DE leads to important complications that could affect the normal visual function and quality of life without treatment [**1**,**6**,**7**]. Most of the standard tests currently used for DE diagnosis (Schirmer test, tear break-up time, fluorescein staining) are not sensitive enough [**8**,**9**]. The introduction of new biomarkers could be helpful, but they could not be used as routine analysis because of some difficulties in their measurement.

Common ophthalmological problems are found in patients with eyelid tumors and include ocular surface diseases (such as dry eyes, eyelid disorders, excessive tearing and ocular inflammation). It was observed that patients with a history of eyelid tumors develop dry eye syndrome and the symptoms and signs are more accentuated than in normal individuals.

## Materials and Methods

This study was conducted in compliance with good clinical practice, institutional review board regulations, informed consent regulations and the principals of the Declaration of Helsinki. The following represented the inclusion criteria for the participants: women with natural menopause and non-Sjögren’s dry eye, ≥ 45 years of age. Subjects were considered PM if they had no menses for at least 12 months. Subjects were diagnosed with DE based on the following: 1) documented diagnosis from medical charts made by a medical care provider ≥ 6 months prior to study visit and 2) a documented history for ≥ 3 months of complaints of ocular discomfort consistent with DE. Exclusion criteria for all subjects included: males, < 45 years of age, childbearing potential or menses within the last 12 months, surgical removal of ovaries with or without fallopian tube, removal of uterus or endometrial ablation, a medical diagnosis of Diabetes and/ or autoimmune connective tissue disease, Stevens-Johnson syndrome, keratorefractive ocular laser procedures, use of topical ocular medications, corneal surgery, punctal cauterization or current punctal plugs. Also, the patients with a history of contact lens wearing within the past 6 months or intraocular laser procedures within 1 year of the study visit were excluded. These patients were classified as normal (NDE) subjects. After providing an informed consent, sixty-six patients were enrolled in the study, divided into two groups of 33 patients each, one group having dry eye disease (DE) and one control group (non-dry eye) (NDE). Associated disorders were also assessed, trying to find out whether there were any correlations between DE and these comorbidities. Thus, the choice of evaluating the eyelid tumor disorders (such as basal cell carcinoma, spinous cell carcinoma and sebaceous carcinoma) as a parameter was made. These patients did not receive cytostatic or surgical treatment. As a result, the two groups of subjects were divided into 4 subgroups, as it follows: NDE non-tumoral, NDE tumoral, DE non-tumoral and DE tumoral.

**Clinical Assessment**

Once enrolled in the study, on the day of collection, subjects responded to dry eye symptom questionnaire (OSDI). Participants completed the questionnaire containing 12 items measuring the visual function, ocular irritation symptoms and the effects of stressful environmental conditions [**11**,**12**].

**Sample Collection and Processing**

The collection of unstimulated tears with a capillary tube took place immediately after slit lamp biomicroscopy. Using a graded disposable 5 μl microcapillary tube (Wiretol-Micropipettes, Drummond Scientific Co., Broomall, PA, USA) of up to 5 μl of tears/ eye was collected from the inferior temporal tear meniscus of each subject, without corneal anesthesia, taking care to ensure that the lid margin and corneal surface were not touched. Later on, tear lysozyme, lactoferrin and lipocalin concentrations were determined via electrophoresis.

After this test, patients underwent routine clinical workup to further characterize disease presence and severity including the assessment of tear breakup time (TBUT), ocular surface fluorescein staining, based on the NEI/ Industry workshop method [**10**]. Schirmer test with and without anesthesia was performed [**13**].

Criteria for diagnosis of DE included an OSDI score >20 with one or more of the following signs: TBUT ≤ 10 seconds, punctate corneal fluorescein staining or Schirmer without anesthesia (Schirmer I) score <10 mm [**13**].

**Tear biomarkers for dry eye disease**

By using Hyrys–Hydrasys SEBIA France automated system, agarose gel electrophoresis substantially enhanced the resolution and the sensitivity of the analysis, despite the fact that SDS polyacrylamide gel electrophoresis gave information about the major tear proteins [**17**].

Tear lipocalin (15–33% of the mass of protein in tears) was a 17.45 kDa member of a family named lipocalins. Tear lipocalin is the major lipid carrier in human tears and is vital to functions involving lipids in the protection of the ocular surface. ~75 μM tear lipocalin is one of the two most concentrated proteins in human tears, along with lysozyme. Tear lipocalin captures phospholipids and fatty acids at the corneal surface, hence avoiding desiccation [**15**]. Because tear lipocalin integrates into the Meibomian lipids, there are implications on the film stability and delay of evaporation at the aqueous-lipid-air interface [**14**,**16**]. Lipocalin may also give a natural protection for fungal infections [**16**].

Lactoferrin (24–27% of total tear proteins), a multifunctional singular chain polypeptide with anti–inflammatory, bacteriostatic and antioxidant properties and lysozyme (44–47%), a glycolytic enzyme with antimicrobial function [**18**] are among the most important peaks that can be identified on SEBIA electropherograms [**17**]. These two proteins, secreted by the acini of the main gland, provide good information about a lacrimal gland malfunction, becoming an indicator of its activity. Furthermore, lower levels are a good signal for an inflammatory answer, reduced antioxidant function and also the predilection for microbial infections (especially lysozyme) [**17**].

## Results

For the statistical analysis, the ANOVA analysis was used. A descriptive analysis (means, medians, standard deviations and range for continuous data and frequency analysis for categorical data) was performed for all the target variables. Continuous quantitative variables were presented as averages +/- standard deviations, while categorical variables were presented as variables. In order to compare continuous variables, the ANOVA analysis was employed. A p value < 0.05 was considered statistically significant. Bivariate correlation analysis (Spearman or Person correlation coefficient calculation) was used to validate the association between the presence of eyelid tumors and dry eye disease.

Thirty-three PM patients with DE were enrolled in this study (mean age 66 years), with a selection based on the inclusion criteria of a recent diagnosis (signs and symptoms) of DE. Thirty-three normal, NDE PM subjects (mean age 62 years) not using artificial tears or lubricants, were recruited as described in the Methods.

**Fig. 1:**
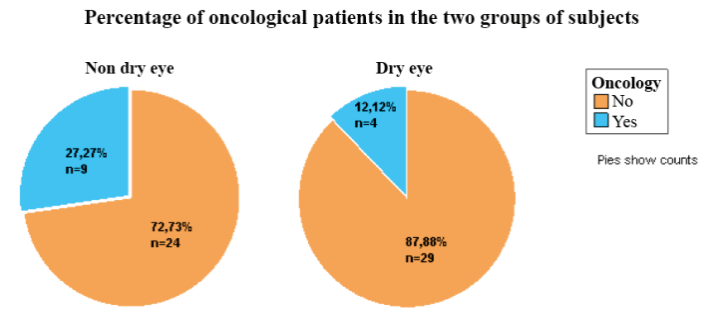
Shows the percentage of oncological patients in the two groups of patients: 27.27% of the NDE patients and 12.12% of the DE patients

From the two groups of patients, 19.70% (13 from 66) of the total number of females had eyelid tumors; patients with oncological pathology in the two major groups represented 27.27% of the NDE patients and 12.12% of the DE patients (**Fig. 1**).

**Fig. 2:**
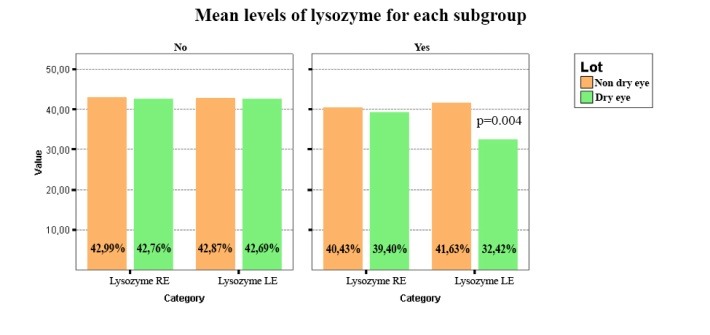
It was found that there is a statistically significant difference between the presence/ absence of eyelid tumors in the DE group for lysozyme (p=0.004). Thus, the concentrations of lysozyme were lower in the presence of eyelid tumors in the DE group, compared to the patients without eyelid tumors from the same group. Also, it was observed that there were lower concentrations for lysozyme in the group with DE, compared with the NDE group in the presence of eyelid tumors

The analysis of variance (ANOVA) showed that there is a statistically significant difference between the presence/ absence of eyelid tumors in the DE group for lysozyme (p=0.004) (**Fig. 2**). Thus, the concentrations of lysozyme were lower in the presence of eyelid tumors in the DE group, compared to the patients without eyelid tumors from the same group. Also, it was observed that there were lower concentrations for lysozyme in the group with DE, compared with the NDE group in the presence of eyelid tumors.

**Fig. 3:**
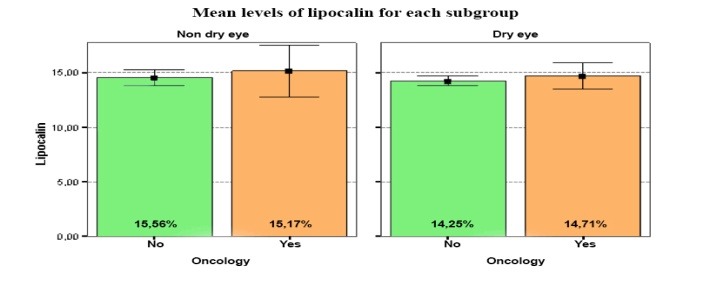
From the statistical data, no correlation was discovered within the lipocalin levels, between the patients with or without eyelid tumors in the NDE group. Thus, tear lipocalins were in the same range in both groups

From the statistical data, no correlation was discovered within the lipocalin concentrations between the patients with or without eyelid tumors in the NDE group. Thus, tear lipocalins were in the same range in both groups (**Fig. 3**).

**Fig. 4:**
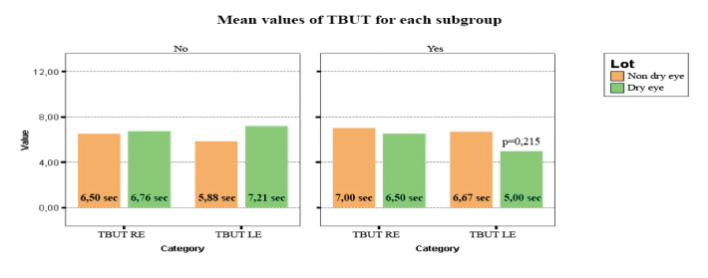
Lower levels for TBUT in the DE group were observed, compared to the NDE group in the presence of eyelid tumors (p=0,215)

Also, lower values for TBUT in the DE group were observed, compared to the NDE group, in the presence of eyelid tumors (**Fig. 4**). 

Although the difference was not statistically significant, it was found that the observation was important in order to establish a connection between the presence of the eyelid tumors and DE.

**Fig. 5:**
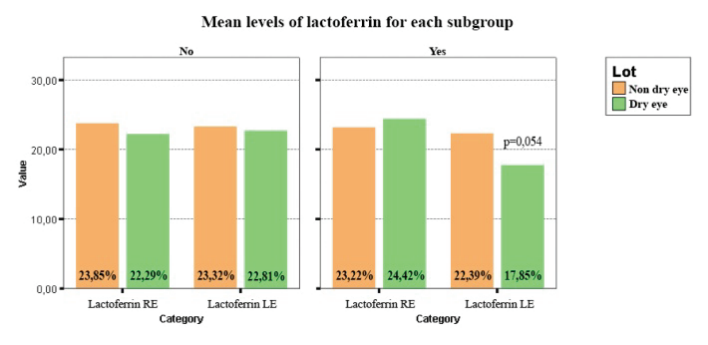
Shows lower concentrations of lactoferrin for the patients with dry eye compared to the patients without dry eye in the presence of eyelid tumors (p=0.054)

Regarding lactoferrin, lower concentrations for the patients with DE were discovered compared to the patients without dry eye in the presence of eyelid tumors (**Fig. 5**).

Even though the difference was not statistically significant, it was considered that this information was relevant in order to find a relation between the presence of eyelid tumors and DE.

The final results containing each test analyzed for the DE group are included in **Table 1**.

**Table 1:** Final results for the DE group

		**ANOVA Table**					
			Sum of Squares	df	Mean Square	F	Sig.
TBUT RE* Oncology	Between Groups	(Combined)	,235	1	,235	,017	,897
	Within Groups		430,310	31	13,881		
	Total		430,545	32			
TBUT LE* Oncology	Between Groups	(Combined)	17,120	1	17,120	1,605	,215
	Within Groups		330,759	31	10,670		
	Total		347,879	32			
Lactoferrin RE* Oncology	Between Groups	(Combined)	16,080	1	16,080	,954	,336
	Within Groups		522,362	31	16,850		
	Total		538,442	32			
Lactoferrin LE* Oncology	Between Groups	(Combined)	86,370	1	86,370	3,995	,054
	Within Groups		670,129	31	21,617		
	Total		756,499	32			
Lyzozyme RE* Oncology	Between Groups	(Combined)	39,571	1	39,571	2,026	,165
	Within Groups		605,352	31	19,527		
	Total		644,922	32			
Lyzozyme LE* Oncology	Between Groups	(Combined)	370,367	1	370,367	9,605	,004
	Within Groups		1195,414	31	38,562		
	Total		1565,782	32			
Lipocalin RE* Oncology	Between Groups	(Combined)	23,968	1	23,968	,527	,473
	Within Groups		1409,822	31	45,478		
	Total		1433,790	32			
Lipocalin LE* Oncology	Between Groups	(Combined)	38,200	1	38,200	1,041	,316
	Within Groups		1137,890	31	36,706		
	Total		1176,090	32			

## Discussion

The evolution of the eyelid tumors was curled and could be accompanied by serious complications, with a possible threat to visual and also vital prognosis of the patient. Thus, any approach to assess this pathology was considered a very important step in the long-term control of this disease, all the steps leading to the obtaining of an as good as possible quality of life.

It was noted that patients with a history of eyelid tumors developed DE and that the symptoms and signs were more severe than in normal individuals. This led to the question whether eyelid tumors influence the biochemical changes at the tear level, what types of mechanisms are involved and to what extent this affected the tear biomarkers.

The concentrations of the three tear biomarkers analyzed (lactoferrin, lysozyme and lipocalin) were examined and some differences between the patients with and without eyelid tumors regarding the presence of DE were discovered.

Thus, it was observed that there was a statistically significant difference between the presence/ absence of eyelid tumors in the DE group for lysozyme (p=0.004). The concentrations of lysozyme were lower in the presence of eyelid tumors in the DE group, compared to the patients without eyelid tumors from the same group. Also, it was noticed that there were lower concentrations for lysozyme in the DE group, compared to NDE group in the presence of eyelid tumors. Further, it was discovered that there was no correlation within the lipocalin concentrations between the patients with or without eyelid tumors in the NDE group. With regard to lactoferrin, lower concentrations for the patients with DE were observed, compared to the NDE patients in the presence of eyelid tumors. Also, it was noticed that there were lower values for TBUT in the DE group, compared to the NDE group in the presence of eyelid tumors. Though the differences were not statistically significant, it was considered that the results were relevant in order to find a relation between the presence of eyelid tumors and DE.

Papers published by Suksri Chotikavanich in 2009 [**19**] and Shinwu Jeong in 2012 [**20**] showed a relation between increased MMP 9 and DE. Also, in 2013, Nick Di Girolamo [**21**] and in 2008, Maya K. Thosani [**22**] found correlations between eyelid tumors and increased MMP activity. Thus, the hypothesis was that there was a correlation between DE and eyelid tumors through the activity of MMP and the assumption that the levels of lysozyme and lactoferrin were launched and are related to the activity of MMP.

These results concluded that there were changes in the biochemical structure and components of the tears of patients having eyelid tumors, compared to normal patients, which led to the development of DE or the enhancement of the symptoms of an existing DE. The amplitude of these changes was correlated with the severity of the disease. This study could help to better assess the patients with eyelid tumors and offer a complete diagnosis and treatment for an underlying condition that may not be obvious, but that may interfere with the quality of life and visual acuity of the patients.

In conclusion, this is a comprehensive study of lipocalin, lactoferrin and lysozyme in dry eye PM women with eyelid tumors in which it was emphasized that electrophoresis of the tear film proteins by using the Hyrys - Hydrasys SEBIA France automated system could be an important tool for the early diagnosis of the tear film changes and prevention of DE.

In order to emphasize the relation between the levels of tear biomarkers and the activity of MMP, further studies should be conducted.

**Acknowledgements**

This work received financial support through the project entitled “CERO – Career profile: Romanian Researcher”, grant number POSDRU/159/1.5/S/135760, cofinanced by the European Social Fund for Sectoral Operational Programme Human Resources Development 2007-2013.

Prof. Corin Badiu is gratefully acknowledged for the constructive criticism and advice during the preparation of the manuscript.

**Sources of funding**

This work received financial support through the project entitled “CERO – Career profile: Romanian Researcher”, grant number POSDRU/159/1.5/S/135760, cofinanced by the European Social Fund for Sectoral Operational Programme Human Resources Development 2007-2013.

**Disclosures**

None
